# Expression of Hormones’ Receptors in Human Corneal Endothelium from Fuchs’ Dystrophy: A Possible Gender’ Association

**DOI:** 10.3390/jcm13133787

**Published:** 2024-06-27

**Authors:** Maria De Piano, Irene Abicca, Valentin Dinu, Anna Maria Roszkowska, Alessandra Micera, Domenico Schiano-Lomoriello

**Affiliations:** 1 Research and Development Laboratory for Biochemical, Molecular and Cellular Applications in Ophthalmological Science, IRCCS-Fondazione Bietti, 00184 Rome, Italy; maria.depiano@fondazionebietti.it; 2 IRCCS-Fondazione Bietti, 00184 Rome, Italy; irene.abicca@fondazionebietti.it (I.A.); domenico.schiano@fondazionebietti.it (D.S.-L.); 3 Department of Ophthalmology, University of Medicine and Pharmacy “Carol Davila”, 050474 Bucharest, Romania; valentin.dinu@umfcd.ro; 4 Department of Biomedical Sciences, Ophthalmology Clinic, University of Messina, Via Consolare Valeria 1, 98124 Messina, Italy; aroszkowska@unime.it; 5 Ophthalmology Department, Faculty of Medicine and Health Sciences, Andrzej Frycz Modrzewski University, 30-705 Krakow, Poland

**Keywords:** hormone, fuchs endothelial corneal dystrophy (FECD), endothelium, cornea, corneal dystrophy, extracellular matrix (ECM), innate response

## Abstract

**Background**: Age and sex are the most significant risk of factors for advanced Fuchs dystrophy. Nevertheless, few data are available on the hormone’s receptor pattern expressed in adult and advanced fuchs endothelial corneal dystrophy (FECD). We investigated the impact of gender, growth factors and extracellular matrix (ECM) regulatory proteins expressed by the dystrophic endothelia. **Methods**: Ten dystrophic endothelial tissues and 10 normal endothelial sheets (corneoscleral specimens; Eye Bank) were used for this characterization study. Hormones’ receptors (ERα, AR, PR, SHBG), few growth factors (VEGFA, βNGF, TGFβ1), some ECM regulators (MMP1, MMP7) and few inflammatory cytokines (IFNγ, IL10) were analyzed by real-time RT-PCR. **Results**: ERα transcripts were significantly increased, AR and SHBG transcripts were decreased in Fuchs endothelia from female patients, and no changes were detected for PR transcripts. VEGFA, βNGF and TGFβ1 transcripts were upregulated in Fuchs’ endothelia, but not significantly linked to gender. High MMP1 and low MMP7 transcripts’ expression were detected in Fuchs’ specimens, mainly in males than females. An increased IFNγ (Th1) transcript expression was observed in females than males, and a trend to increase for IL10 (Th2) transcripts was detected in males than females. **Conclusions**: Our findings clearly indicate that hormone receptors, growth factors and matrix mediators as well as a Th1 pathway are predominant in Fuchs’ dystrophy, displaying a pattern of expression specific for the female phenotype. The differential expression of hormones’ receptors and the Th1/Th2 ratio might prompt to new theories to be tested in vitro and in vivo models, such as the use of hormonal substitute for counteracting this endothelial cell lost.

## 1. Introduction

Fuchs’ Endothelial Corneal Dystrophy (FECD, herein shorted as Fuchs) is a corneal disease characterized by the progressive degeneration of corneal endothelial cells, leading to the abnormal deposition of ECM components (mainly fibronectin and type-1 collagen), which consist in excrescences in Descemet’s membrane known as corneal guttae [1,2,3,4,5]. Consisting of a single layer of hexagonal-shaped endothelial cells, the corneal endothelium plays an essential role in maintaining corneal transparency through the control of stromal hydration [6,7]. The endothelial impairments slowly prompt corneal edema formation and lead to loss of vision in the absence of corneal transplantation [5]. Nowadays two main techniques of corneal surgery are available, known as the posterior lamellar techniques, the Descemet’s Stripping Automated Endothelial Keratoplasty (DSAEK) or the Descemet Membrane Endothelial Keratoplasty (DMEK) [8].

The incidence of Fuchs is higher in women (3–4:1 female/male ratio) [2,9,10,11]. The pathogenesis of Fuchs is not completely understood. Genetic and environmental factors are involved in the pathogenesis of Fuchs. Age and sex are the most significant risk factors for advanced Fuchs dystrophy [2,9]. Inflammatory and the oxidative stress mediators have been considered the main actors involved in the loss of corneal endothelial cells, providing a new therapeutic target [12].

ECM overt production occurs by an impaired regulation of proteases (matrix metalloproteinases, MMPs) and related inhibitors (tissue inhibitors of MMPs; TIMPs) that in turn are modulated by Vascular Endothelial Growth Factor-A (VEGF-A), Nerve Growth Factor (NGF), Transforming Growth Factor β (TGFβ) and a specific Th1/Th2 ratio [13,14,15]. Particularly, anti-inflammatory and profibrogenic factor are also recognized as crucial regulators of the endothelial to mesenchymal transition (EndMT), as observed in several models and Fuchs’ tissues [16,17]. As well, the expression of NGF and VEGF-A receptors by corneal endothelial cells prompt to some homeostatic, modulating and healing abilities displayed by endothelial cells [18].

In recent years, a special interest has been devoted to the associations between the ocular system and hormones (androgens, estrogens and progesterone) [19]. The current concept is that sex hormones can drive cell differentiation and survival outside the endocrine systems and particularly at the ocular surface [19]. Besides the well-known effect on tear production, estrogens (ER) also induce the release of pro-inflammatory mediators (TNFα, IL1β and IL6) from cornea and conjunctiva, and stimulate locally the immune response, contributing to the development of dry eye syndrome [20]. The influence of sexual hormones on both cornea and conjunctiva, as well as their association with the inflammatory processes in some corneal pathologies, as corneal dystrophies, are still not well known. Female sexual hormones can influence female healthy cornea in term of corneal thickness and curvature under physiological conditions (ovulation, pregnancy and breastfeeding) [21,22]. Overall, these data suggest a strong association between corneal thickness and female hormones. Some recent literature data suggest an influence of sex hormones on the evolution and severity of corneal pathologies, but no data are available on the correlation between the expression of corneal hormone receptors and inflammation in Fuchs’ dystrophy. 

Herein, we sought to investigate the effect of gender on the expression of hormones receptors in the corneal endothelium of Fuchs’ affected patients. The expression of estrogen receptor alpha (ERα), androgen receptor (AR), progesterone receptor (PR) and the steroid carrier protein Sex Hormone-Binding Globulin (SHBG) were quantified. We also correlate these receptors with the expression of growth factors, as VEGF, NGF, TGFβ1, metalloproteinases (MMP1, MMP7), regulators of ECM protein production, and some inflammatory cytokines (INFγ and IL10) known to play a crucial role in the survival of corneal endothelium. 

## 2. Materials and Methods

### 2.1. Ethical Considerations and STUDY Population

The study population included patients referred to the Anterior Segment Unit of IRCCS Fondazione Bietti (Rome, Italy) between January 2020 to October 2021, with a diagnosis of Fuchs Dystrophy based on slit lamp examination, the presence of guttae, and corneal edema requiring corneal endothelial surgery [23]. Sample collection included the dystrophic endothelium and the normal corneal endothelium from the remaining corneoscleral ring (donor) provided by the Eye Bank (San Giovanni, Rome, Italy). Exclusion criteria were: the presence of any corneal disease (such as herpetic keratitis or stromal scar, and/or history of previous corneal, refractive, glaucoma, or retinal surgery); the diagnosis of glaucoma and the use of topical anti-glaucomatous therapy; previous complicated cataract surgery and/or anterior chamber intraocular lens implants or surgical aphakia; history of anti-VEGF intraocular injections, corneal, intraocular and/or systemic diseases in the 3 month prior to surgery of infectious origin; systemic neurodegenerative diseases (Alzheimer’s, Parkinson’s); autoimmune diseases (such as Sjögren’s syndrome) or oncological ones; intra/post-operative complications of corneal endothelial surgery. The study was conducted in accordance with the ethical standards stated in the Declaration of Helsinki. The study was approved by the intramural committee (Ifo/Bietti; Prot.88/19/FB). Written informed consent was obtained from patients before their enrollment in the study and before clinical and biostrumental data collection and samples’ management. Only patients requiring therapeutic corneal endothelial transplantation were enrolled for the study. 10 endothelial tissues from Fuchs dystrophy (3 male/7 female) and 10 age and sex representative normal corneoscleral derived endothelial tissues (control; 5M/8F, corneoscleral rings from Eye Bank, San Giovanni, Rome, Italy) were collected at the end of surgery and quickly stored in medium and delivered to the laboratory for further stabilization at −80 °C. 

### 2.2. Clinical Assessment and Routine Biological Tests

**Figure 1:**
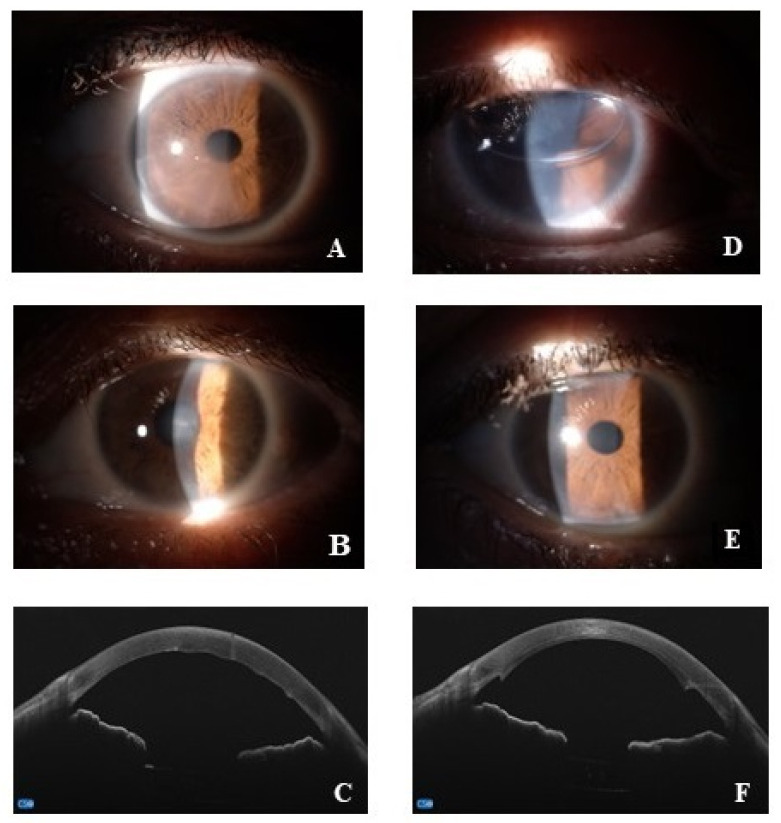
Representative pre (**A**–**C**) and post (**D**–**F**) surgery acquisitions. (**A**–**C**) anterior segment images at slit lamp (**A**,**B**) and OCT acquisitions of corneal Fuchs (**C**); (**D**–**F**), anterior segment digitally acquired images at 1-day (**D**) and 1-month (**E**) post-surgery; anterior segment OCT at 1-month post-surgery (**F**).

All patients underwent a preoperative visit including general and ocular anamnesis, and a complete ophthalmological evaluation, with the best-corrected distance visual acuity (BCVA), the slit lamp biomicroscopic examination, the applanation tonometry (Goldmann-tonometer), the real-time endothelial cell density (ECD). As Central Corneal Thickness (CCT) also represents an important parameter used by clinicians to monitor Fuchs disease, cornel pachymetry was performed using an anterior segment-OCT microscope combined with Placido-disk corneal topography (MS-39, CSO, Florence, Italy). As suggested by recommendation rules, preoperative pachymetry measurement threshold ranged from 600 to 640 μm [24]. Finally, the preoperative visit also included a general medical examination and blood leukocyte count, proteinogram, sera electrolytes (sodium, potassium and calcium), renal/hepatic function and coagulation factors as well as urine tests. Representative slit lamp and OCT acquisitions are shown in Figure 1.

### 2.3. Surgical Procedures

All patients included underwent an ultrathin DSAEK, performed according to the Price and Price method (2008) by the same skilled surgeon (D.S.L) [25]. Donor corneoscleral tissue was prepared by the Eye Bank using an automated microkeratome (Moria, Antony, France) with an intended anterior lamellar thickness of 300/350 μm, and sent to the surgical unit in stabilizing medium (Eusol-C, Alchimia, Padova, Italy) [26]. 

The application of an anterior chamber maintainer avoids the anterior chamber collapse. The central Descemet Membrane of the dystrophic cornea (host cornea) was stripped under air insufflation and the donor graft (donor folded endothelium) was inserted into the eye using a Macaluso inserter (E. Janach, Como, Italy), through a clear corneal incision and was positioned centrally. Intracameral air or gas (20% SF6) bubble was used to facilitate the fixation of graft to the host cornea. The day after surgery, if necessary, the anterior chamber was refilled with gas. Patients with intraoperative complications were excluded from the study.

### 2.4. Molecular Analysis: Tissue Processing and 2-Step Real-Time RT-PCR Analysis

Total RNA was extracted in Trifast reagent solution (1:1; EuroClone, Milan, Italy), and normalized RNA samples (100 ng/sample) were used to synthesize cDNA using the IMPROM kit and random primers (Promega, Madison, WI, USA) in a conventional termocycler (LifePRO/BIOER, Euroclone, Milan, Italy). Specific amplifications were carried out using one-intron spanning pair primers properly designed for the following target/referring genes specific for: hormonal receptor pattern (AR, PR, ERα, SHBG), growth factors (VEGFA, βNGF, TGFβ1), matrix enzymes (MMP1, MMP7), cytokines (INFγ and IL10) and housekeeping (H3, GAPDH). Information on primer sequences and amplicons’ length are reported in Table 1. Normalized cDNAs were amplified by using a SYBR green hot-start PCR mastermix (Hydra Taq; Biolab, Biocell, Rome, Italy) in a 48-well microplate real-time PCR platform (Eco™ Illumina, San Diego, CA, USA). Specific amplifications were tested by verifying the single curve specific for each amplicon. The amplification protocol included the following: pre-hold (5 min at 50 °C) and pre-incubation for 15 min at 95 °C. Each of the 39 amplification cycles consisted of 30 s/94 °C denaturation, followed by a specific annealing step at 58–60 °C and 30 s/72 °C extension. Annealing was set at appropriate temperatures Ta = (Tm–5 °C) and verified for specificity via grading if required. The melting curve was registered from 56.0 °C to 94.1 °C (0.3 °C hold for 00:00:01 between reads). Single melting curves were verified at the end of each amplification, cycle threshold (Ct) values were detected, and target gene expressions were provided by software (row data) according to the 2-(ΔΔCt) formula (ΔΔCt = ΔCt sample − ΔCt calibrator). The single-target gene expressions (fold changes, FC) were expressed in the log2 scale, as directly provided by Illumina software with respect to the emmetropic group (normal values). REST 384–2006 software was also used to estimate changes in transcripts’ expression, as calculated with respect to two referring genes (H3 and GAPDH).

### 2.5. Biochemical Analysis: SDS-PAGE and IntDen Analysis

Total proteins were extracted from endothelial tissues according to a standard procedure (Trifast reagent solution (1:1; EuroClone, Milan, Italy) and were eluted in 2x Loading-Buffer (Invitrogen, Waltham, MA, USA) supplemented with β-mercaptoethanol. Elutions were boiled (98 °C/5 min) and loaded on 4–20% SDS-PAGE minigels (miniprotean; Biorad, Hercules, CA, USA). After separation, gels were stained according to a standard procedure (SYPRO Ruby gel stain; Thermo Fisher, MA, USA) and acquired in a B-BOX Blue Light LED epi-Illuminator (Smobio, Hsinchu City, Taiwan). The free available ImageJ v1.43 was used for the Integrated Optical Density (IntDen) analysis. Band quantification was carried out on 8-bit TIFF converted digital images. IntDen data were calculated for each band, grouped (mean ± SD) and subjected to statistical analysis.

### 2.6. Statistical Analysis

The distribution for molecular analysis (2log expression) was analyzed using Kolmogorov-Smirnov test, confirming the assumption of records coming from a normally distributed population (Prism vs. 10.0.0; GraphPad Software Inc., San Diego, CA, USA). The One-way ANOVA coupled Kruskal-Wallis test post hoc was used to estimate differences between groups. Significant levels are indicated in the panels with asterisks (*p* value summary: * *p* ≤ 0.05; ** *p* ≤ 0.005; *** *p* ≤ 0.0005; **** *p* ≤ 0.0001). All data were provided as mean ± SEM. 

## 3. Results

A total of 20 corneal endothelial specimens (10 from Fuchs dystrophy 2M/8F and 10 from related control specimens’ age/sex-matched) were analyzed for transcriptomic expression. The residual tissues of donor corneas not used during the corneal transplant operation were used as controls. The main ocular and demographic data are summarized in Table 2.

All females were in menopause and none of them were on hormone replacement therapy. Two females were in levothyroxine treatment for non-autoimmune hypothyroidism. Hypercholesterolemia was diagnosed in 5 patients (4F/1M), and 3 out 4F were in therapy with statins. Seven patients (6F/1M) were affected by hypertension whereas 5 patients (4F/1M) were in therapy with anti-aggregating/anticoagulant formulations. None of the patients had a diagnosis of neurodegenerative diseases. Any patient was a smoker in the preoperative period. 

### 3.1. PR, ERα, AR and SHBG Transcripts’ Expression in Fuchs Endothelia

**Figure 2:**
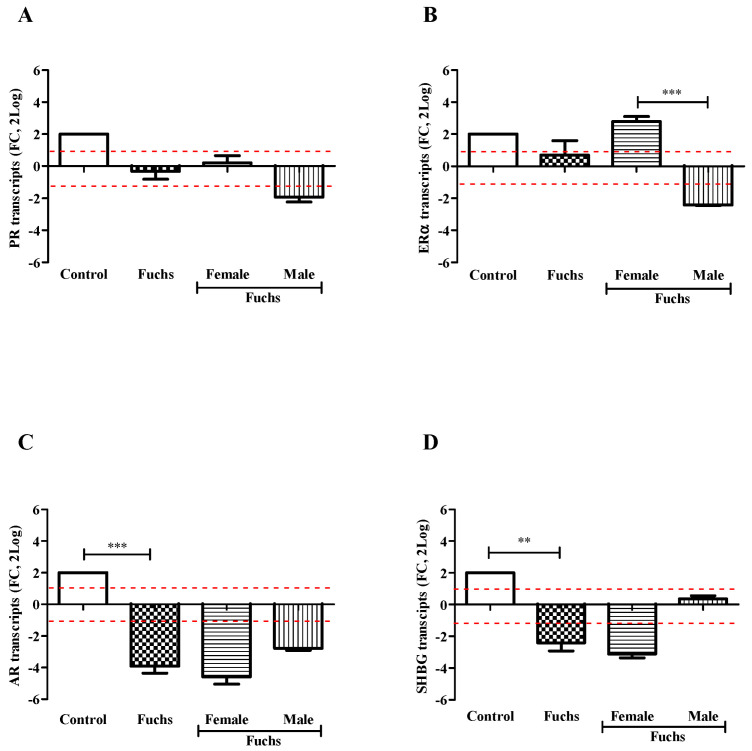
Hormones expression and gender analysis. Endothelial tissues were extracted and subjected to real-time RT-PCR. Differences between Fuchs, Female and Male affected by Fuchs are shown for PR (**A**), ERα (**B**), AR (**C**), and SHBG (**D**) and indicated by asterisks, as calculated using Kruskal-Wallis test. Note the significant difference in mRNAs specific for ERα (**B**) between sex and decrease for AR (**C**) and SHBG (**D**) in Fuchs vs. Control. Data are 2log-FC (fold changes, ±SEM), as calculated with respect to Controls and referred to as 1 (white box). Red-dotted lines indicate the level of significance for relative PCR. Significant levels are shown as calculated using Kruskal-Wallis test (** *p* ≤ 0.005; *** *p* ≤ 0.0005).

Control endothelial specimens express basal transcript levels for all targets investigated. As shown in Figure 2, the transcription of targets specific for PR (A), ERα (B), AR (C) and SHBG (D) receptors was reduced in Fuchs endothelia, as compared to control ones. The case-to-control analysis showed that the decreased target gene expression was most evident in Female specimens for AR (C) and SHBG (D) with respect to PR (A) and ERα (B). Gender analysis showed that PR transcripts (A) were unchanged in Fuchs’ dystrophy (*p* > 0.05); ERα transcripts (B) were significantly upregulated in Females (*p* < 0.0005) with respect Males; and finally, the expressions of AR (C, *p* < 0.0005) and SHBG (D, *p* < 0.005) transcripts were significantly deregulated in Fuchs disease vs. control.

A preliminary protein analysis on the same samples confirmed the changes of PR (138,593.75 ± 6506.94 IntDen in female vs. 118,545.60 ± 1935.35 IntDen in male; *p* > 0.05) and ERα (158,386.00 ± 660.49 IntDen in female vs. 112,416.50 ± 546.45 IntDen in male; *p* > 0.05) receptors’ protein. 

### 3.2. Fuchs-Endothelia Synthesizes Growth Factors and Matrix Enzymes’ Transcripts

**Figure 3:**
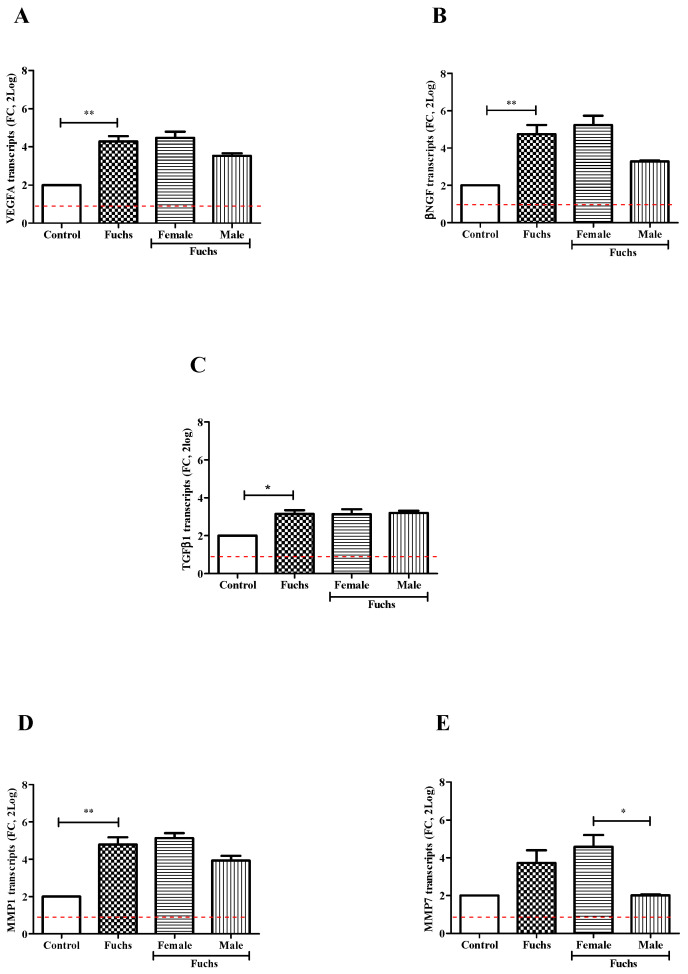
Growth and ECM remodeling modulators’ expression and gender analysis. Fuchs-affected corneal endothelia synthesize transcripts specific for growth factors VEGFA (**A**), βNGF (**B**) and TGFβ1 (**C**)) and matrix enzymes MMP1 (**D**) and MMP7 (**E**) that were analyzed using relative real-time RT-PCR. A significant transcript expression was observed for VEGFA (**A**), βNGF (**B**), TGFβ1 (**C**) and MMP1 (**D**) in patients affected by Fuchs vs. Control and for MMP7 (**E**) in Females vs. Males. Data are 2log-FC (fold changes, ±SEM), as calculated with respect to Controls and referred to as 1 (white box). Red-dotted lines indicate the level of significance for relative PCR. Significant levels are shown as calculated using Kruskal-Wallis test (* *p* ≤ 0.05; ** *p* ≤ 0.005).

In Figure 3, VEGFA (A, *p* < 0.005), βNGF (B, *p* < 0.005) and TGFβ1 (C, *p* < 0.05) transcripts’ expressions were significantly upregulated in Fuchs-affected specimens as compared to control ones. By contrary, gender analysis showed a trend toward an increase for expression of VEGFA (A), βNGF (B) and TGFβ1 (C) transcripts in Females vs. Males. Besides the overall expression of TGFβ1 transcripts was also significantly increased in Fuchs-endothelia (*p* < 0.05), the transcription appeared not depending on gender (C). Therefore, to better characterize TGFβ1 phenotype, the expression of two matrix enzymes (MMP1 and MMP7) involved in growth factors’ activation was also investigated. As shown in Figure 3, a consistent high expression of MMP1 transcripts (D, *p* < 0.005) occurred in Fuchs-affected specimens and a low expression of MMP7 transcripts (E, *p* < 0.05) was detected in Males with respect to Female ones.

### 3.3. Fuchs-Endothelia Display a Th1 Phenotype

**Figure 4:**
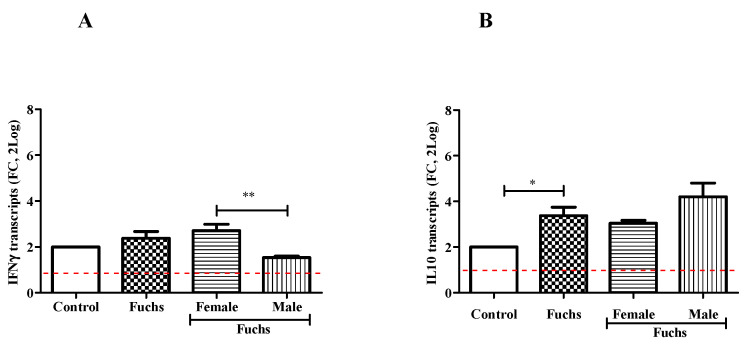
Th1 expression profile and gender analysis. The molecular analysis showed a Th1 phenotype for Fuchs population: IFNγ mRNA (**A**) expression was increased in Female population as compared to Males. IL10mRNA (**B**) expressions were increased in Fuchs specimens as compared to Control and referred to as 1 (white box; red-dotted line). Significant levels are shown as calculated using Kruskal-Wallis test (* *p* ≤ 0.05; ** *p* ≤ 0.005).

As shown in Figure 4, the analysis of cytokine pattern displayed changes in IFNγ content between Females and Males (A; *p* < 0.005), while an increased expression of IL10 levels was observed in Fuchs-affected endothelia specimens (B, *p* < 0.05).

### 3.4. Correlation Analysis

AR correlated positively with ERα (r = 0.890, *p* = 0.043) and MMP7 (r = 0.918, *p* = 0.010), look like ERα with MMP7 (r = 0.966, *p* = 0.008). βNGF correlated negatively with PR (r = −0.836, *p* = 0.010). 

## 4. Discussion

The contribution of sexual hormones in the pathogenesis of many degenerative diseases has been understudied for years, and only recently gender influence has been prospected as one of the possible ways to improve a target therapy [27,28,29]. Regarding the ocular tissues, it is well known that some female predominance can influence the developing, ongoing and exacerbation of some anterior segment associated ocular diseases, such as dry eye syndrome [30]. Recently, female predominance was also reported in age-related macular degeneration (AMD) and linked to the levels of complement factors that vary by sex, implying that sex can be one of the main risk factors for AMD development [31]. 

One of the key features of Fuchs’ dystrophy is the female prevalence [11,32]. Regarding the pathogenesis, few studies highlighted the possible toxicity of estrogen metabolites to corneal endothelium, as observed in vitro studies [33,34]. Previous data on ER expression in corneal endothelium were scarce and inconsistent [35]. Some interesting works pointed at the expression of hormones receptors [35,36]. Very recently, further studies emphasized that the estrogen genotoxicity can cause preferential development of Fuchs’ endothelial corneal dystrophy in females [33,34]. Although a possible hormonal role has been postulated so far and the association of Fuchs dystrophy with female phenotype is still undoubted, none was able to explain the mechanisms behind the development of Fuchs’ dystrophy and its predominance in females [37]. 

Our findings clearly indicate that hormone receptors, growth factors and matrix enzymes as well as a Th1 pathway are predominant in Fuchs’ dystrophy and particularly in the female phenotype. 

First, we observed that the transcription of ERα is significantly increased in Fuchs corneal endothelial specimens from female patients, while the transcripts’ expression of AR and SHBG targets were significantly decreased, and no changes were detected for PR. At least for PR and ERα, the preliminary biochemical analysis on single tissues confirmed the molecular data. This finding is interesting as all women were in menopause and without hormonal treatment, implying the involvement of additional factors working in Fuchs’ dystrophy.

The fact that VEGFA (angiogenic factor), βNGF (neurotrophic factor) and TGFβ1 (anti-inflammatory and profibrogenic factor) were upregulated in Fuchs’ dystrophy but not significantly linked to gender, is in line with previous studies [38,39]. Although NGF is more expressed in male than female, no significant changes were observed in the expression at the ocular levels except for adolescents or women in sexual activity [40]. 

As known, Fuchs’ dystrophy is linked to ECM remodeling and apoptotic signals [13]. No data are available on the potential effect of ECM-modulator MMP7 also known to activate βNGF. The similar expression pattern between βNGF and MMP7 might support the hypothesis of an involvement of this growth factor in the neuroprotection occurring in female tissues. While the association of TGFβ1 and IL10 was reported in Fuchs’ dystrophy, our data suggest that these profibrogenic and anti-inflammatory mediators are not dependent by sex phenotype. Outside of any explanation is the Th1-IFNγ association with female phenotype and the Th2-IL10 association with male phenotype.

There are few weaknesses associated with the present study. The main limitation is related to the limited number of samples that does not allow us to strengthen the results of our study and the number of specimens (a monolayer of endothelial cells). In addition, the limited number of endothelial cells obtained from these Fuchs specimens does not yield enough total protein to use alternative techniques such as Western blotting or protein chip array. Current studies are in progress to implement this weak point.

The corneal endothelium is a monolayer of hexagonal cells lining the posterior surface of anterior chamber that is in charge for corneal transparency due a constant pumping of ions and is in continuous exposure to aqueous humor [41]. The influence of aqueous signature cannot be ignored and might regulate the endothelial function [38]. Indeed, the expression of ERα, AR and PR in corneal endothelial cells might be modulated by circulating hormones (estrogen, androgen and progesterone status), a key determinant of endothelial cell function and stress response (iNOS release) in healthy pre/post-menopausal women [42].

## 5. Conclusions

Fuchs is an age-related, genetically heterogeneous, oxidative stress disorder resulting in loss of endothelial cells, mainly via apoptosis, and progressive damage of the corneal structure (loss of transparency, edema), that has been frequently associated with female phenotype [11]. The contribution of sexual hormones in the pathogenesis of many degenerative diseases of the eye is an understudied topic gaining increasing interest as the sex differences account for the prevalence and outcomes of several age-related eye disorders [43]. Taken together, our study prompt to a different expression of hormones’ receptors in Fuchs endothelia between female and males, supporting the knowledge that gender represents a risk factor for late Fuchs dystrophy appearance. Our findings clearly indicate that changes in hormone receptors, growth and matrix mediators as well as a Th1 pathway are predominant in Fuchs’ dystrophy and particularly in a female phenotype. There is little known about how hormones levels can alter the behavior of endothelial cells and Th1/Th2 ratios, despite evidence that ageing can effectively influence local immunity and vasculogenic aspects. The meaning of this expression is under investigation as this correlate with growth factors and tissue remodeling mediators.

Overall, our findings have some clinical implications, as the corneal transplantation is the unique treatment available. Endothelial cells are exposed to adverse changes during lifetime (reproductive period and menopause/andropause), and their vulnerability to hormonal changes cannot be misjudged [44]. Therefore, a better understanding of cellular and molecular mechanisms regulating endothelial cell behavior upon in vitro hormone exposure of aged cells might clarify some apoptotic aspects. The outcomes of hormone replacement trials might suggest potential reinforcement of the endothelial monolayer and possibly lower the entity of cellular apoptosis.

## Figures and Tables

**Table 1:** Primers’ details.

Referring Gene	Forward and Reverse Sequences	Accession Number
H3	F: 5′-GTC TGC AGG CTG GCA TAG AAG-3′ R: 5′-TCG CCT TCT GGG TTG AGT G-3′	NM005324.4
18S	F: 5′-GGA GAG GGA GCC TGA GAA C-3′ R: 5′-AGG GCC TCG AAA GAG TCC T-3′	NR003286
Target gene		
PR	F: 5′-TAC GGA GCC AGC AGA AGT CC-3′ R: 5′-TGA AGC TCT CAG TCC CTC GC-3′	X51730.1
ERα	F: 5′-GTG GTG CCC CTC TAT GAC CT-3′ R: 5′-TGC CTC CCC CGT GAT GTA AT-3′	X03635
AR	F: 5′-GGG GAC ATG CGT TTG GAG AC-3′ R: 5′-CTG TTT CCC TTC AGC GGC TC-3′	M34233
SHBG	F: 5′-AAA TCA CTC CCT CTG GGT CC-3′ R: 5′-AAG TCA AGA TGG AGG GGG AC-3′	BC112186.1
VEGF A	F: 5′-TGA CAG GGA AGA GGA GGA GA-3′ R: 5′-CGG TGT TCC CAA AAC TGG-3′	AF022375.1
βNGF	F: 5′-TGA AGC TGC AGA CAC TCA GG-3′ R: 5′-CAC CTC CTT GCC CTT GAT GT-3′	BC126150.1
TGFβ1	F: 5′-GAG ATG AGG GTT TCC ACG AG-3′ R: 5′-GCG CCG AGA TGT AGT TAT CC-3′	BC017288
MMP1	F: 5′-TCC CAG AGA GCA GCT TCA GT-3′ R: 5′-CCT ATC CAG GGT GAC ACC AG-3′	BC013875
MMP7	F: 5′-GAG CTC ATG GGG ACT CCT AC-3′ R: 5′-ACT GCT ACC ATC CGT CCA G-3′	BC003635
IFNγ	F: 5′-ACC TAA GCA AGA TCC CAT GGG-3′ R: 5′-TGG GTA CAG TCA CAG TTG TCA A-3′	NM_000619.3
IL-10	F: 5′-GCC TGA CCA CGC TTT CTA GC-3′ R: 5′-GGC TCC CTG GTT TCT CTT CC-3′	M57627

Primers (accession number) were designed with one intron spanning (https://bioinfo.ut.ee/primer3-0.4.0/, accessed on 10 August 2005) from human mRNA complete sequences available at https://www.ncbi.nlm.nih.gov/gene (accessed on 10 August 2005) with 50% to 60% GC content and >61 °C annealing temperature.

**Table 2:** Demographic and ocular characteristics (central corneal thickness and Phakia/Pseudo-Phakia). Legend: M, male; F, female; CCT (µm) = central corneal thickness.

Pt.	Gender	Age	CCT (µm)	Phakia/PseudoPhakia
1	F	70	601	Phakia
2	F	84	945	Phakia
3	F	73	599	Combined PHACO + IOL
4	F	71	669	Combined PHACO + IOL
5	M	69	693	Previous PHACO + IOL
6	M	88	616	Combined PHACO + IOL
7	F	76	745	Previous PHACO + IOL
8	F	70	750	Previous PHACO + IOL
9	F	57	538	Combined PHACO + IOL
10	F	78	659	Phakia

## Data Availability

The data that support the findings of this study are available from the corresponding author upon reasonable request. Patients or the public were not involved in the design, or conduct, or reporting, or dissemination plans of our research.
